# Supplementary material for: Multi‐dataset identification of innovative feature genes and molecular mechanisms in keratoconus
Source: J Cell Mol Med. 2024 Sep 19;28(18):e70079. doi: 10.1111/jcmm.70079 (PMC11412914; doi:10.1111/jcmm.70079)
Supplement: Supplementary file 2 — Material S2. [file JCMM-28-e70079-s002.zip › 7.GSEA analysis/5.RNF217-AS1/KEGG_B_CELL_RECEPTOR_SIGNALING_PATHWAY.html]

Details for gene set KEGG\_B\_CELL\_RECEPTOR\_SIGNALING\_PATHWAY[GSEA]

|  || Dataset | RNF217-AS1.group.cls#h\_versus\_l |
| Phenotype | group.cls#h\_versus\_l |
| Upregulated in class | h |
| GeneSet | KEGG\_B\_CELL\_RECEPTOR\_SIGNALING\_PATHWAY |
| Enrichment Score (ES) | 0.22769721 |
| Normalized Enrichment Score (NES) | 0.9152829 |
| Nominal p-value | 0.6023622 |
| FDR q-value | 1.0 |
| FWER p-Value | 1.0 |
Table: GSEA Results Summary

  

Fig 1: Enrichment plot: KEGG\_B\_CELL\_RECEPTOR\_SIGNALING\_PATHWAY      
 Profile of the Running ES Score & Positions of GeneSet Members on the Rank Ordered List

  

| SYMBOL | TITLE | RANK IN GENE LIST | RANK METRIC SCORE | RUNNING ES | CORE ENRICHMENT || 1 | CD79B | na | 162 | 0.680 | 0.0571 | Yes |
| 2 | NFATC2 | na | 219 | 0.642 | 0.1137 | Yes |
| 3 | PIK3R5 | na | 1058 | 0.434 | 0.1289 | Yes |
| 4 | CD19 | na | 1121 | 0.425 | 0.1657 | Yes |
| 5 | PIK3AP1 | na | 1140 | 0.424 | 0.2037 | Yes |
| 6 | PPP3R2 | na | 1532 | 0.389 | 0.2277 | Yes |
| 7 | LILRB3 | na | 3340 | 0.277 | 0.2007 | No |
| 8 | IKBKB | na | 4368 | 0.238 | 0.1927 | No |
| 9 | CHP2 | na | 5296 | 0.206 | 0.1847 | No |
| 10 | PIK3CD | na | 6778 | 0.159 | 0.1564 | No |
| 11 | RAC3 | na | 7163 | 0.148 | 0.1588 | No |
| 12 | DAPP1 | na | 8024 | 0.127 | 0.1455 | No |
| 13 | VAV2 | na | 9125 | 0.103 | 0.1231 | No |
| 14 | SOS1 | na | 9467 | 0.096 | 0.1219 | No |
| 15 | BLNK | na | 10819 | 0.073 | 0.0895 | No |
| 16 | GSK3B | na | 10962 | 0.071 | 0.0919 | No |
| 17 | CD79A | na | 11168 | 0.068 | 0.0921 | No |
| 18 | PIK3CB | na | 11439 | 0.064 | 0.0901 | No |
| 19 | KRAS | na | 11445 | 0.064 | 0.0957 | No |
| 20 | NFATC1 | na | 11693 | 0.060 | 0.0940 | No |
| 21 | NFAT5 | na | 12924 | 0.043 | 0.0624 | No |
| 22 | RAC2 | na | 12945 | 0.043 | 0.0657 | No |
| 23 | MAPK1 | na | 13362 | 0.038 | 0.0572 | No |
| 24 | PPP3CB | na | 13986 | 0.031 | 0.0420 | No |
| 25 | PIK3CA | na | 14021 | 0.031 | 0.0438 | No |
| 26 | MAPK3 | na | 14127 | 0.029 | 0.0434 | No |
| 27 | AKT2 | na | 15816 | 0.011 | -0.0043 | No |
| 28 | HRAS | na | 15878 | 0.010 | -0.0052 | No |
| 29 | PIK3R1 | na | 16145 | 0.007 | -0.0122 | No |
| 30 | MAP2K2 | na | 16236 | 0.006 | -0.0142 | No |
| 31 | CHUK | na | 16613 | 0.003 | -0.0248 | No |
| 32 | NRAS | na | 17304 | -0.005 | -0.0443 | No |
| 33 | AKT1 | na | 18280 | -0.013 | -0.0712 | No |
| 34 | RAC1 | na | 18357 | -0.014 | -0.0721 | No |
| 35 | NFKBIB | na | 18425 | -0.015 | -0.0727 | No |
| 36 | INPP5D | na | 18494 | -0.015 | -0.0733 | No |
| 37 | BTK | na | 18772 | -0.018 | -0.0797 | No |
| 38 | RAF1 | na | 18969 | -0.020 | -0.0835 | No |
| 39 | SOS2 | na | 19710 | -0.027 | -0.1024 | No |
| 40 | NFKBIE | na | 19736 | -0.028 | -0.1006 | No |
| 41 | NFKB1 | na | 20531 | -0.035 | -0.1203 | No |
| 42 | IKBKG | na | 20686 | -0.037 | -0.1215 | No |
| 43 | PPP3R1 | na | 20845 | -0.038 | -0.1225 | No |
| 44 | CHP1 | na | 21142 | -0.042 | -0.1273 | No |
| 45 | FCGR2B | na | 21452 | -0.045 | -0.1322 | No |
| 46 | RELA | na | 21550 | -0.046 | -0.1308 | No |
| 47 | CD72 | na | 21868 | -0.049 | -0.1355 | No |
| 48 | GRB2 | na | 22374 | -0.055 | -0.1451 | No |
| 49 | PIK3R2 | na | 22894 | -0.061 | -0.1546 | No |
| 50 | MAP2K1 | na | 23215 | -0.064 | -0.1580 | No |
| 51 | PPP3CA | na | 24064 | -0.076 | -0.1756 | No |
| 52 | NFATC3 | na | 24080 | -0.076 | -0.1691 | No |
| 53 | SYK | na | 24484 | -0.082 | -0.1732 | No |
| 54 | PTPN6 | na | 24509 | -0.083 | -0.1664 | No |
| 55 | CD81 | na | 24658 | -0.085 | -0.1629 | No |
| 56 | NFKBIA | na | 24662 | -0.085 | -0.1553 | No |
| 57 | VAV3 | na | 24686 | -0.086 | -0.1482 | No |
| 58 | PLCG2 | na | 24806 | -0.087 | -0.1437 | No |
| 59 | LYN | na | 24892 | -0.089 | -0.1381 | No |
| 60 | FOS | na | 25888 | -0.105 | -0.1573 | No |
| 61 | MALT1 | na | 26345 | -0.113 | -0.1601 | No |
| 62 | PPP3CC | na | 26944 | -0.124 | -0.1661 | No |
| 63 | JUN | na | 27259 | -0.131 | -0.1633 | No |
| 64 | BCL10 | na | 28485 | -0.162 | -0.1840 | No |
| 65 | CARD11 | na | 28816 | -0.171 | -0.1780 | No |
| 66 | NFATC4 | na | 29771 | -0.199 | -0.1874 | No |
| 67 | PIK3CG | na | 30335 | -0.218 | -0.1839 | No |
| 68 | CD22 | na | 31102 | -0.246 | -0.1837 | No |
| 69 | AKT3 | na | 31832 | -0.281 | -0.1792 | No |
| 70 | PIK3R3 | na | 32895 | -0.352 | -0.1778 | No |
| 71 | CR2 | na | 33316 | -0.387 | -0.1548 | No |
| 72 | IFITM1 | na | 34009 | -0.484 | -0.1309 | No |
| 73 | VAV1 | na | 34083 | -0.499 | -0.0877 | No |
| 74 | PRKCB | na | 34175 | -0.520 | -0.0431 | No |
| 75 | RASGRP3 | na | 34495 | -0.650 | 0.0067 | No |
Table: GSEA details [plain text format]

  

Fig 2: KEGG\_B\_CELL\_RECEPTOR\_SIGNALING\_PATHWAY      
 Blue-Pink O' Gram in the Space of the Analyzed GeneSet

  

Fig 3: KEGG\_B\_CELL\_RECEPTOR\_SIGNALING\_PATHWAY: Random ES distribution      
 Gene set null distribution of ES for **KEGG\_B\_CELL\_RECEPTOR\_SIGNALING\_PATHWAY**
